# Supplementary material for: Identification and Structural Elucidation of Anti-Inflammatory Compounds from Chinese Olive (Canarium Album L.) Fruit Extracts
Source: Foods. 2019 Sep 26;8(10):441. doi: 10.3390/foods8100441 (PMC6836117; doi:10.3390/foods8100441)
Supplement: Supplementary file 1 [file foods-08-00441-s001.pdf]

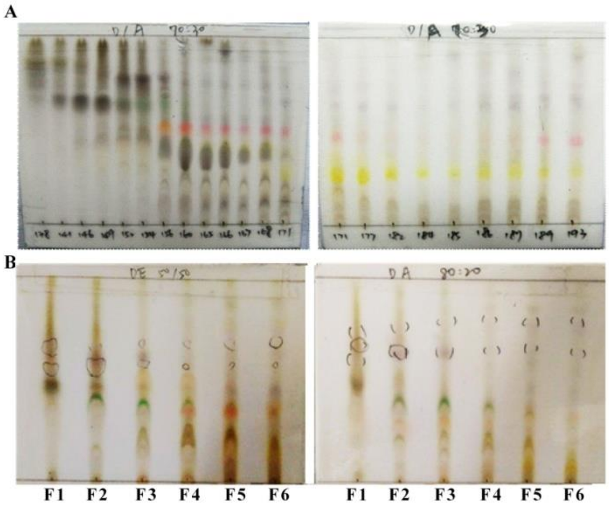

Figure S1. TLC result of subfractions from Fr. F. (A) Fractions before merge (mobile phase is Acetone (A) / Dichloromethane (DCM)= 3:7, v/v) (B) Fractions after merge (left TLC plate, mobile phase is Ethyl acetate (EA)/DCM = 1:1, v/v; right TLC plate, mobile phase is A/DCM= 2:8, v/v).

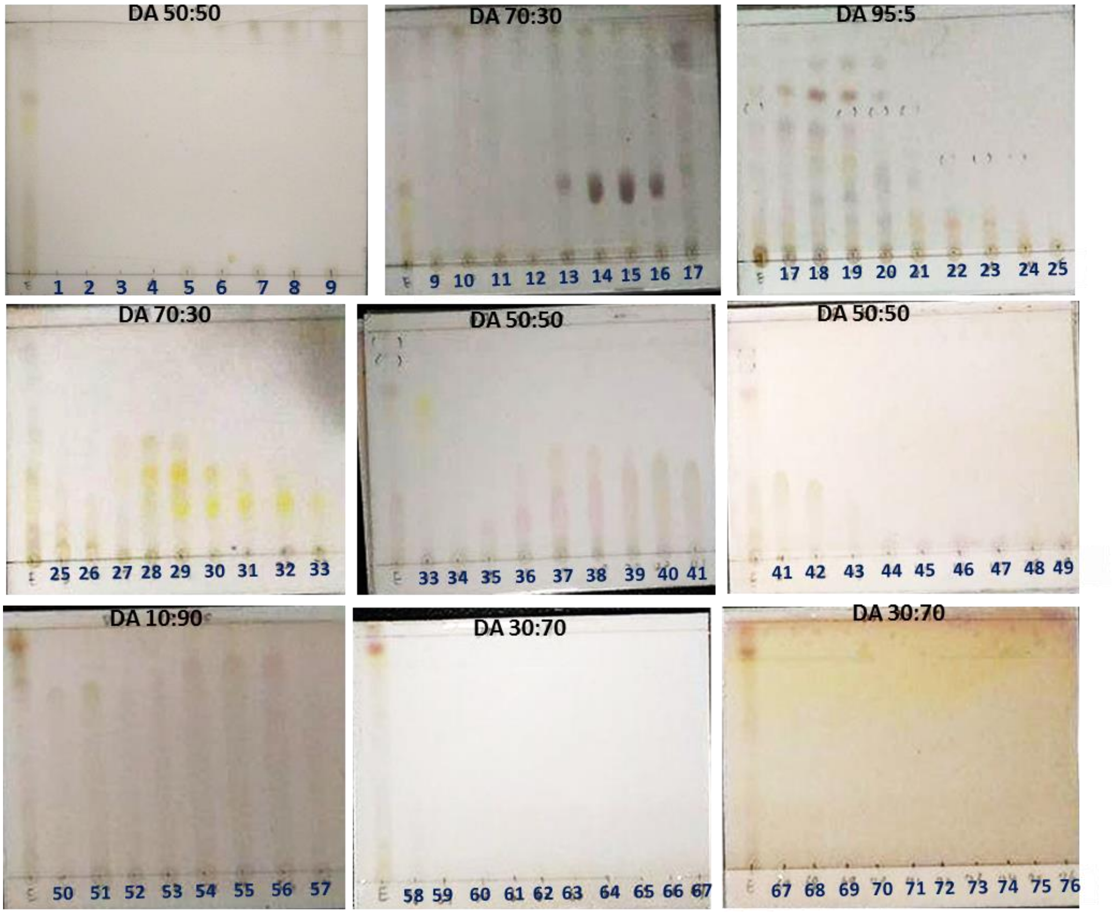

Figure S2. TLC result of subfractions from Fr. F5. Fractions before merge (mobile phase is labeled on the top of TLC plate)

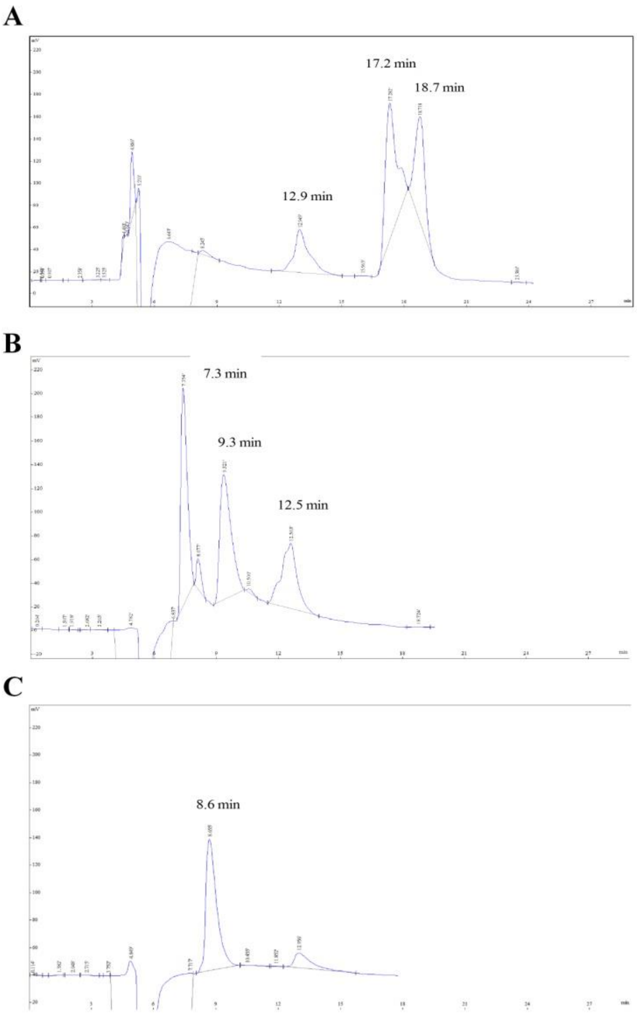

Fig. S3. The HPLC chromatogram. (A) HPLC chromatogram of Fr. F5b. (B) HPLC chromatogram of Fr. F5e. (B) HPLC chromatogram of Fr. F5f.

A

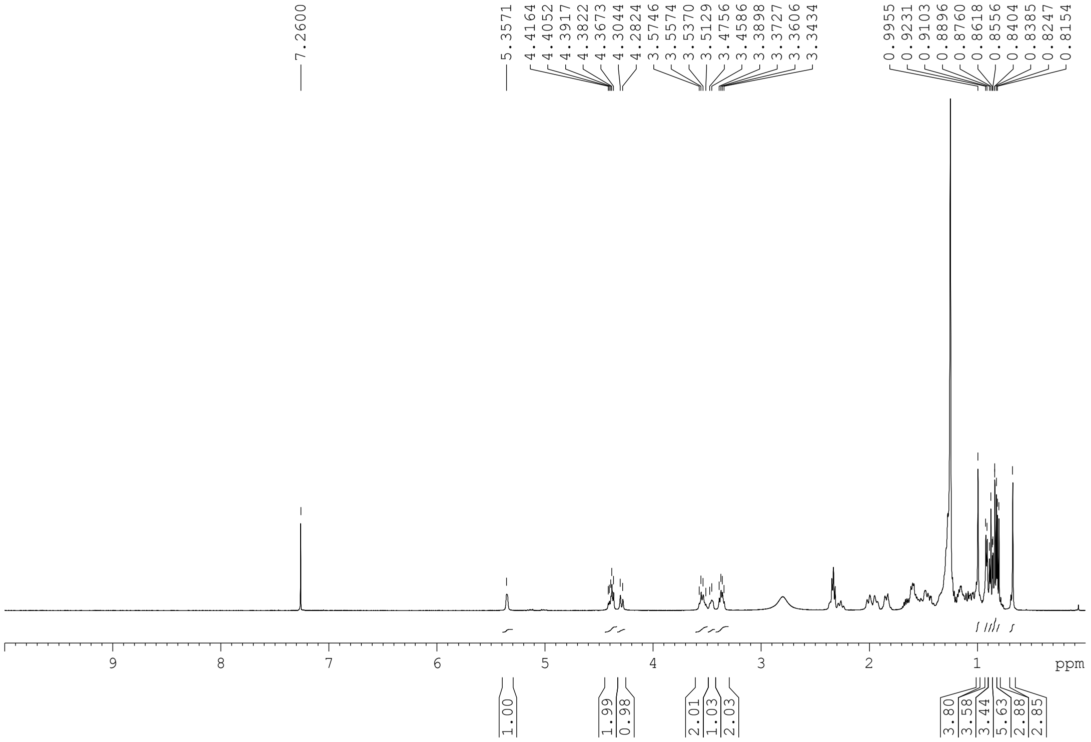

^{1}H NMR spectrum of sitoindoside I in chloroform-*d*.

B

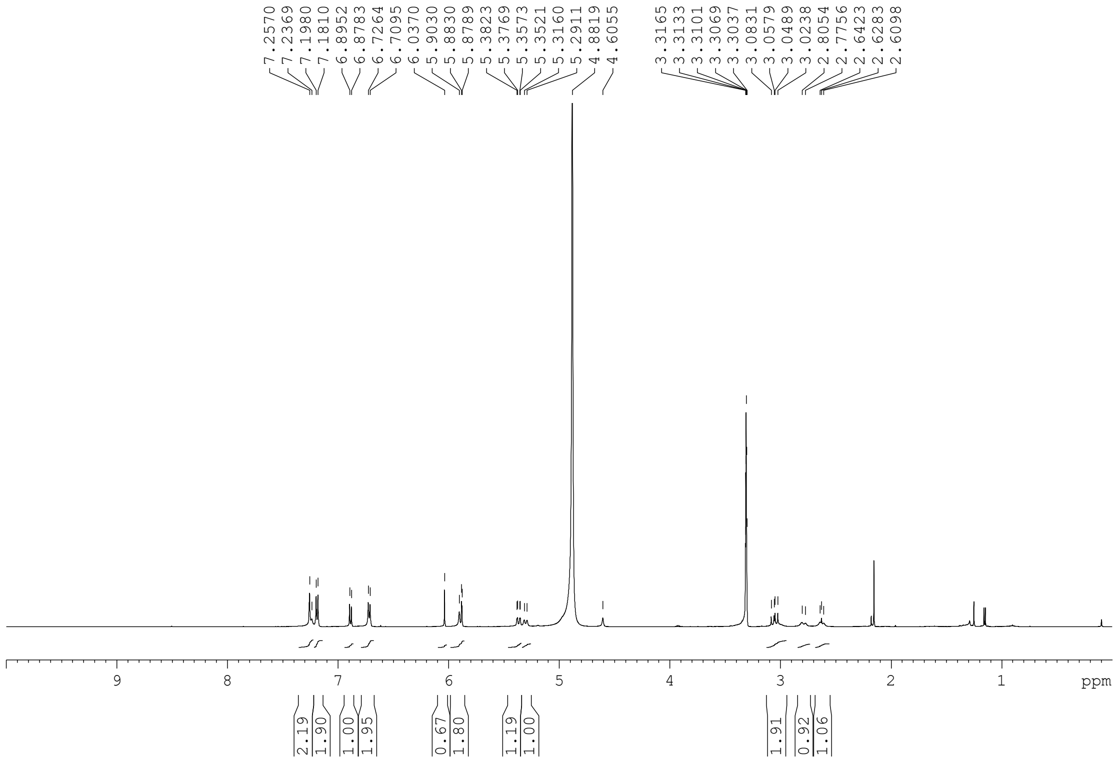

^{1}H NMR spectrum of tetrahydroamentoflavone in methanol-d_4.

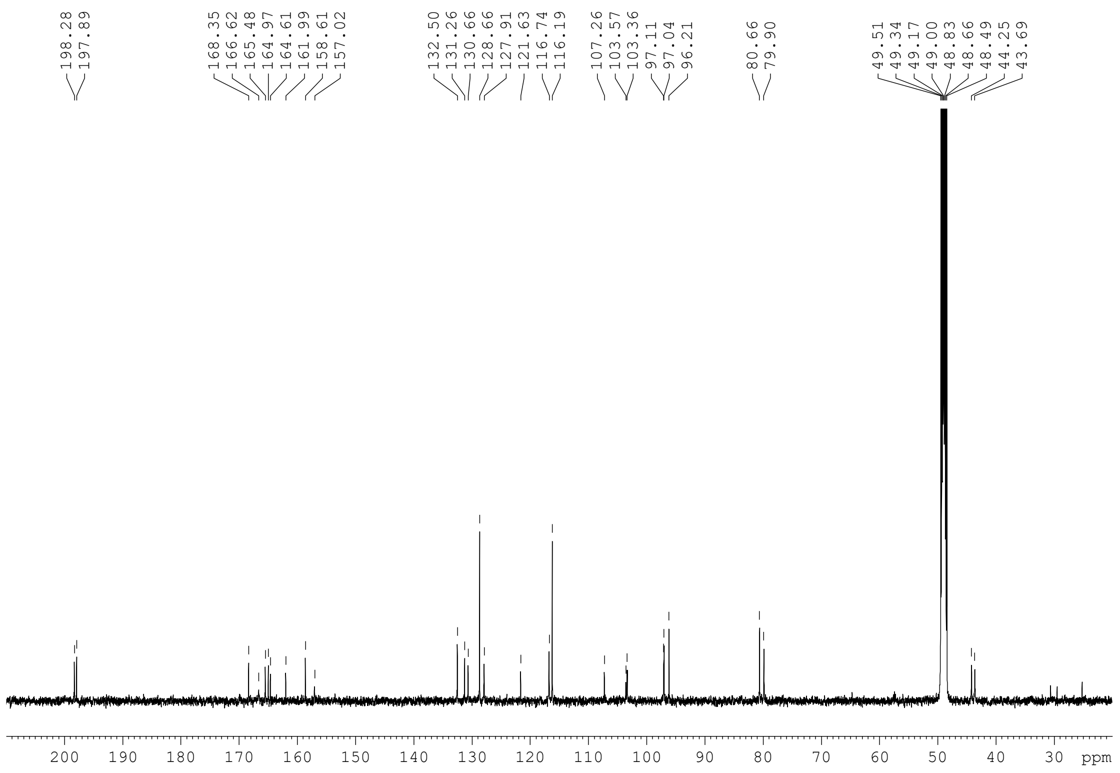

^{13}C NMR spectrum of tetrahydroamentoflavone in methanol-d_4.

C

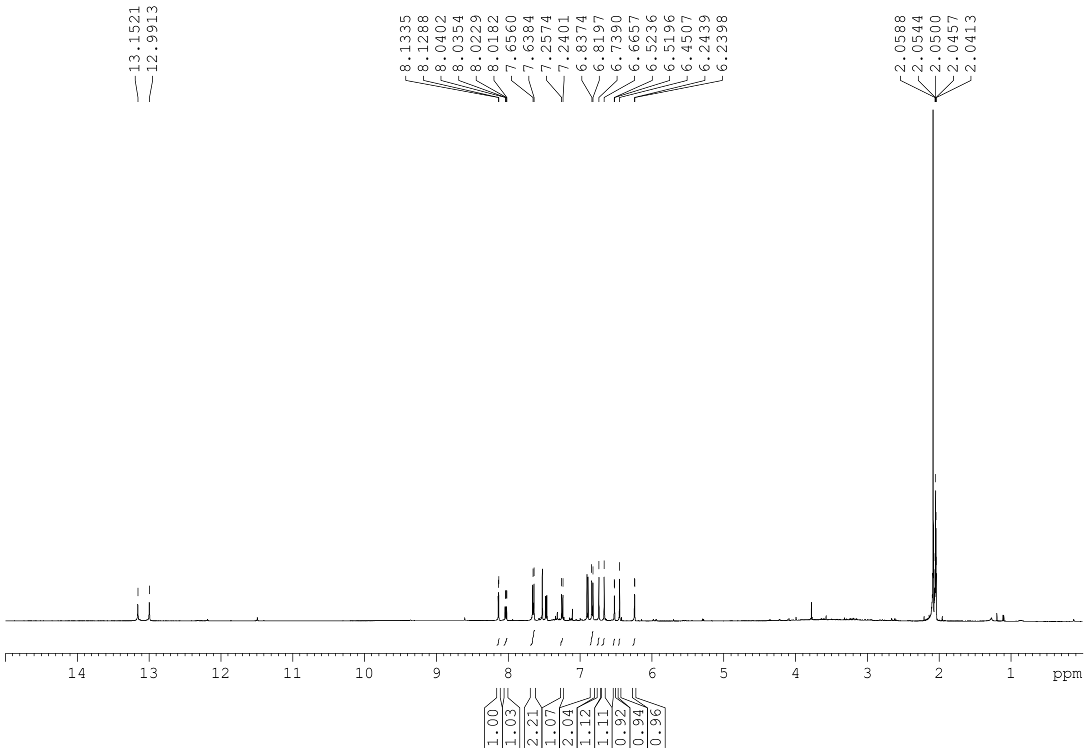

^{1}H NMR spectrum of amentoflavone in acetone-d_6.

D

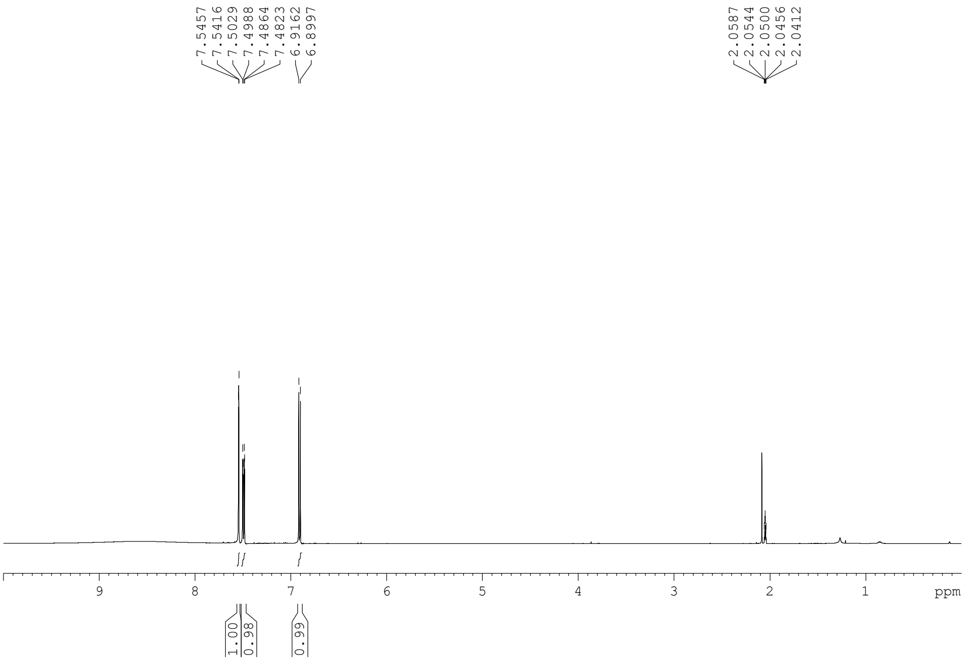

^{1}H NMR spectrum of protocatechuic acid in acetone-d_6.

Fig. S4. The NMR spectrum. (A) ^{1}H NMR spectrum of sitoindoside I in chloroform-*d*. (B) ^{1}H NMR and ^{13}C NMR spectrum of tetrahydroamentoflavone in methanol-d_4. (C) ^{1}H NMR spectrum of amentoflavone in acetone-d_6 (D) ^{1}H NMR spectrum of protocatechuic acid in acetone-d_6.
